# Pulmonary exacerbations and clinical outcomes in a longitudinal cohort of infants and preschool children with cystic fibrosis

**DOI:** 10.1186/s12890-017-0546-8

**Published:** 2017-12-11

**Authors:** Jordana E. Hoppe, Brandie D. Wagner, Scott D. Sagel, Frank J. Accurso, Edith T. Zemanick

**Affiliations:** 1 Department of Pediatrics, University of Colorado School of Medicine and Children’s Hospital Colorado, 13123 E 16th Avenue, B-395, Aurora, CO 80045 USA; 2 Department of Biostatistics and Informatics, University of Colorado School of Public Health, Aurora, CO USA

**Keywords:** Cystic fibrosis, Pulmonary exacerbation, Antibiotics, Lung function

## Abstract

**Background:**

Pulmonary exacerbations (PEx) in school aged children and adults with cystic fibrosis (CF) lead to increased morbidity and lung function decline. However, the effect of exacerbations in young children with CF is not fully understood. We sought to characterize the frequency and clinical impact of PEx in a pilot study of infants and pre-school aged children with CF.

**Methods:**

Thirty young children with CF [median (range) 1.5 years (0.2–4.9)] were prospectively followed for 2 years. Exacerbation frequency (hospitalizations and outpatient antibiotic use) was determined. Chest radiographs were performed at enrollment and study completion and assigned a Brasfield score. Lung function at age 7 years was assessed in a subset of children. The association between PEx frequency, chest radiograph score, and lung function was determined using Spearman correlation coefficients and corresponding 95% confidence intervals. Correlations with an absolute magnitude of 0.3 or greater were considered clinically significant.

**Results:**

Over 2 years, participants experienced a median of two PEx (range 0–13). Chest radiograph scores at enrollment and study completion were inversely associated with PEx frequency (*R* = −0.48 and *R* = −0.44, respectively). The association between frequency of PEx and lung function [forced expiratory volume in 1 s (FEV_1_)] at age 7 years was small (*R* = 0.20). Higher forced vital capacity (FVC) at 7 years was associated with more frequent PEx during the study (*R* = 0.44).

**Conclusions:**

Children with worse chest radiograph scores had more frequent PEx over the subsequent 2 years, suggesting a group of patients at higher risk for PEx. Frequent PEx in infants and young children with CF were not associated with lower FEV_1_ and FVC at 7 years, although spirometry in this age group may not be a sensitive marker of mild lung disease and disease progression.

**Electronic supplementary material:**

The online version of this article (10.1186/s12890-017-0546-8) contains supplementary material, which is available to authorized users.

## Background

Cystic fibrosis (CF) is a chronic, life-limiting disease characterized by airway inflammation, infection, progressive obstructive lung disease and lung function decline [1]. Lung involvement is the leading cause of morbidity and mortality in patients with CF [2]. It is known in older children and adults that pulmonary exacerbations (PEx) lead to lung function decline [3]. However, the effect of PEx on clinical outcomes in young children with CF is not fully understood. Previous studies have demonstrated that airway inflammation [4, 5] and bronchiectasis [6–8] occur in infants and preschoolers with CF indicating that lung damage occurs early in life. Additionally, newborn screening has led to earlier diagnosis and provides an opportunity for medical interventions to slow the progression of lung disease [9, 10].

Accordingly, we sought to examine the frequency of PEx in a cohort of infants and preschool aged children with CF and the relationship of these exacerbations to clinical outcomes, including Brasfield chest x-ray (CXR) scores and lung function at 7 years. Most PEx in children are treated on an outpatient basis with oral antibiotics [11]; thus, we captured these events as well as hospitalizations. We employed oropharyngeal cultures, the standard of care in the United States for monitoring airway infections in non-expectorating patients, to determine airway microbiology. We hypothesized that those with more frequent PEx during the study period would have worse clinical outcomes at the conclusion of the study (CXR scores) and at school age (lung function).

## Methods

### Study population and design

Subjects between the ages of 3 months and 4 years with a diagnosis of CF based on a sweat chloride ≥60 mEq/L and/or the presence of two known CF mutations were eligible to enroll (see Table 1 for demographic information). Potential subjects were approached and recruited for the study during regularly scheduled clinic visits. Subjects completed study visits at their quarterly CF clinic visits and at the time of clinician defined PEx for 2 years (Additional file 1: Figure S1). The determination of a PEx was based on two criteria: 1) diagnosis by the primary CF provider and 2) initiation of oral or intravenous (IV) antibiotics for respiratory symptoms. A standardized definition of PEx was not used in order to capture all antibiotic courses that were prescribed. However, using criteria from the Inhaled Hypertonic Saline in Infants Study (ISIS), the subjects’ signs and symptoms as well as reasons for treatment with antibiotics were retrospectively analyzed to determine how many of these encounters would have been classified as exacerbations based on standardized criteria [12].

**Table 1:** Subject characteristics at baseline

Demographics	
Female: Male	13:17
Age at first visit in years, median (range)	1.5 (0.2, 4.9)
Genotype	
F508/F508	17 (57%)
F508/Other	10 (33%)
High risk genotype, *n* = 29^a^	28 (96%)
Method of diagnosis^b^	
Diagnosed by newborn screen	25 (83%)
Meconium ileus	5 (17%)
Clinical diagnosis	2 (7%)
Oropharyngeal culture results	
Negative culture	16 (53%)
*Staphylococcus aureus*	11 (36%)
*Pseudomonas aeruginosa*	0 (0%)
*Haemophilus influenzae*	6 (20%)
Weight z-score, median (range), *n* = 29	−0.36 (−2.3, 0.91)
Pancreatic insufficient, *n* = 30	30 (100%)

^a^In one patient a second mutation has not been identified

^b^Three patients were diagnosed by both meconium ileus and a positive newborn screen

At each study visit (both routine clinic visits and exacerbation visits), subjects underwent a history, physical, medication history, height/weight, vital signs including pulse oximetry and an oropharyngeal (OP) swab. OP swabs were processed for comprehensive microbiology following Cystic Fibrosis Foundation consensus guidelines [13]. Cough severity was assessed at each visit by parent and physician report using an ordinal cough score ranging from 0 (no cough) to 3 (frequent) for parent report of day and nighttime symptoms and from 0 (no cough) to 4 (frequent) for cough during physician exam (Wisconsin Cough Score), with a maximum possible score of 10 [14]. Antibiotic data was retrospectively captured at each quarterly visit to assess for antibiotics prescribed by primary care providers, at urgent care visits or through telephone triage.

Chest radiographs were obtained at enrollment and at study completion during periods of clinical stability (defined by lack of increased cough, sputum production, wheezing or other symptoms of PEx) and were scored by the Brasfield system by two trained radiologists [15]. The two chest radiograph scores were averaged to obtain the final score. If subjects were ill at the time of enrollment or study completion, chest radiograph was done at the next well visit.

Lung function data was obtained following study completion through our clinical database for patients who had reached 6.5 years of age. The highest percent predicted forced expiratory volume in 1 s (FEV_1_) and associated forced vital capacity (FVC) value between 6.5 and 7.5 years were selected for analysis [16]. The Colorado Multiple Institutional Review Board approved the study. Written informed consent was obtained for all patients. HIPAA standards were maintained during the study. Portions of this publication have previously been presented in abstract form [17].

### Statistical analysis

This pilot study was powered to detect an effect size of 0.55 standard deviations or larger for the change in chest radiograph scores over 2 years; a sample size of 30 patients is needed to achieve 80% power using a two-tailed 0.05 significance level paired t-test. Descriptive statistics were used to define the baseline demographic characteristics of this cohort. We determined the relationship between factors using Spearman correlation coefficients and corresponding 95% confidence intervals; correlations with an absolute magnitude of 0.3 or greater were considered clinically significant. Average and standard error estimates for Wisconsin cough scores were obtained across study visit type (stable, exacerbation) using an analysis of variance and generalized estimating equations to account for repeated scores within subjects. Percent predicted for follow-up pulmonary function tests were calculated using the Global Lung Function Initiative (GLI) equations. All analyses were performed using SAS version 9.4 software (SAS Institute Inc.: Cary, NC, 2014).

## Results

### Subject characteristics and pulmonary exacerbations

We recruited 30 children (13 females, 17 males) with CF; 4 withdrew prior to the end of the study. Baseline clinical characteristics of study subjects are summarized in Table 1. A total of 99 PEx were recorded and 98 antibiotic courses prescribed over 2 years. Most exacerbations (87%) were treated with oral antibiotics alone. Additional details about the type and class of antibiotics is provided in Table 2. Retrospectively applying the criteria in the Inhaled Hypertonic Saline in Infants Study (ISIS) [12], 98 out of 99 met exacerbation criteria. In the encounter that did not meet these criteria, one patient had been started on antibiotics prior to being seen and at the time of the clinic visit they were asymptomatic with no increased symptoms reported.

**Table 2:** Characteristics of prescribed antibiotic courses

Antibiotic characteristics	
Total number of antibiotic courses	98
Prescribed antibiotics	
*IV*	7 (7%)
*Oral*	85 (87%)
*IV + Oral*	6 (6%)
Antibiotic classes	
*Cephalosporin*	27 (22%)
*Penicillin*	21 (17%)
*Aminoglycoside*	7 (5%)
*Fluoroquinolone*	5 (4%)
*Trimethoprim-sulfamethoxazole*	59 (47%)
*Macrolide*	2 (2%)
*Vancomycin*	2 (2%)
*Carbapenem*	1 (1%)

Twenty-seven of the 30 (90%) children reported at least one PEx (95% CI 73.5% – 97.9%) over the 2 years of the study. Subjects experienced a median of 2 PEx (range 0–13), with no difference by gender [females median (range) 4 (1–7) compared to 2 (0–13) for males]. Age also did not impact the number of PEx; children under 2 years of age at study enrollment had a similar number of PEx compared to those over 2 years [median (range) of 3 (0–13) compared to 2 (0–6)]. Wisconsin cough scores were higher at the time of a PEx [mean estimate (95% CI): 5.1 (4.7, 5.6)] compared to baseline clinic values [1.2 (0.9, 1.6)] and completion of exacerbation treatment [1.0 (0.7, 1.3)]. There was no association identified between number of PEx and the presence of *Staphylococcus aureus* at the time of enrollment or history of meconium ileus (data not shown). Dornase alfa was used by patients enrolled in the study for 57% of visits. No association was observed between the use of dornase alfa and exacerbation frequency [*R* = 0.20, (−0.18, 0.52)].

### Hospitalizations

The majority (85%) of PEx were treated in the outpatient setting; however, 8 children required hospitalization for respiratory symptoms (*n* = 15 hospitalizations, range 1–5 per patient). Hospitalized patients received IV antibiotics 87% of the time (all patients receiving IV antibiotics were hospitalized). One subject did not receive any antibiotics during a hospitalization as symptoms were more consistent with an asthma exacerbation and a second subject only received oral antibiotics as the illness was felt to be consistent with viral bronchiolitis. Viral studies were performed due to a suspected viral respiratory infection in 8 out of the 15 hospitalizations; 5 out of the 8 viral studies were positive [three for a single virus (respiratory syncytial virus, rhinovirus and human metapneumovirus) and two for multiple viruses (coronavirus and enterovirus/rhinovirus; respiratory syncytial virus and human metapneumovirus)]. Of the 8 hospitalized patients, 6 had lung function data available at 7 years of age. No differences were seen in the distribution of FEV_1_ in patients who were hospitalized compared to those subjects who were not hospitalized [median (range): 109% (89–136) vs 102% (92–138)] but the small number of hospitalized subjects limits our ability to make conclusions.

### Chest radiograph scores

**Fig. 1:**
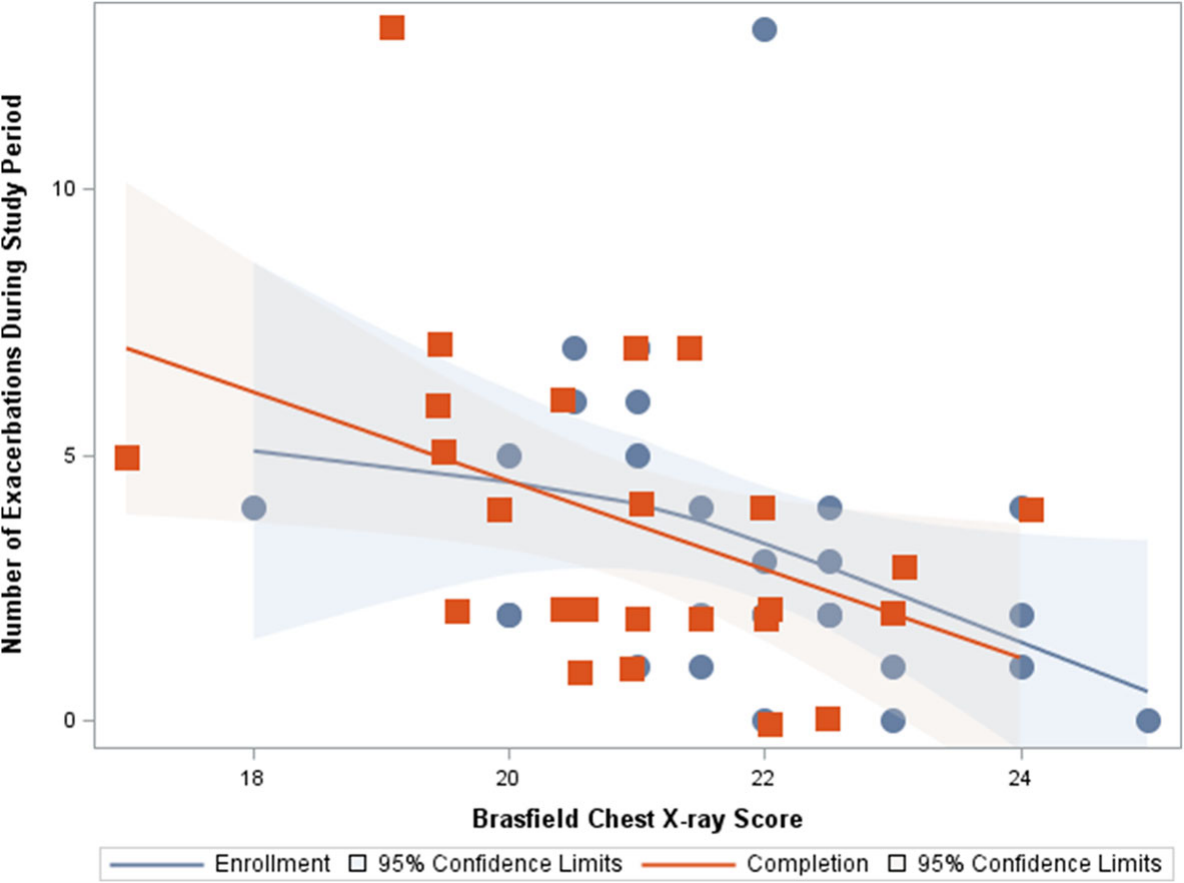
Relationship between chest radiograph score at enrollment and study completion and number of exacerbations: A loess curve (solid line) was used to display the association and is plotted with a 95% confidence interval (shaded band). Chest radiograph scores at enrollment and study completion were associated with the frequency of pulmonary exacerbations, indicating that those with better chest radiograph scores had fewer exacerbations. The Spearman’s coefficient suggests a moderate association

**Fig. 2:**
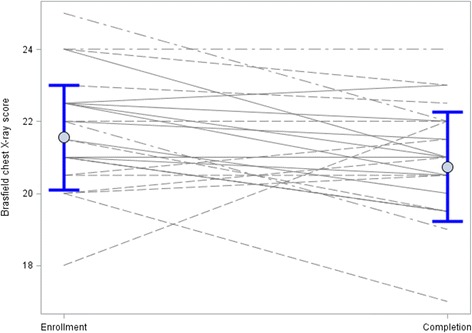
Chest x-ray scores at enrollment and study completion: Chest x-ray scores for individual subjects are depicted. Points and whiskers at each point correspond to means and standard deviation, respectively

Chest radiographs were obtained at the beginning and end of the study in 25 of the 30 subjects (1 declined post-study x-ray and 4 withdrew prior to end of study). Chest radiographs were assigned a Brasfield score, with lower scores indicating worse disease. Chest radiograph scores at enrollment (Fig. 1), [*R* = −0.48, (−0.72, −0.13)] and at study completion, [*R* = −0.44, (−0.70, −0.04)], were associated with the number of PEx during the study with better chest radiograph scores in children with fewer exacerbations. The average chest radiograph score worsened slightly over 2 years with a mean decrease in the Brasfield score of 0.8 (95% CI: -1.4, −0.1) (Table 3). Change in chest x-ray scores for individual patients are depicted in Fig. 2 and details of the subscores are included in the data supplement (See Additional file 2: Table S1).

**Table 3:** Clinical outcomes

Clinical Outcome	Results
Number of exacerbations, median (range)	2 (0,13)
Age at study completion in years, median (range)	3.7 (2.2–7.1)
Brasfield chest x-ray scores, enrollment	21.5 (18.0,25.0)
Brasfield chest x-ray scores, study completion	21.0 (17.0,24.0)
Change in chest x-ray scores, *n* = 25, median (range)	−0.5 (−3.0, 4.0)*
Culture results, n = subjects with positive culture at any time over two-year study period, (%)	
*Staphylococcus aureus*	26 (87%)
*Pseudomonas aeruginosa*	4 (13%)
*Haemophilus influenza*e	29 (97%)
*Stenotrophomonas maltophilia*	4 (13%)
PEx Culture results, n = positive culture during PEx, (% of PEx) [# subjects with positive culture]	
*Staphyloccus aureus*	37 (39%) [18]
*Pseudomonas aeruginosa*	2 (2%) [2]
*Haemophilus influenzae*	27 (28%) [15]
*Stenotrophomonas maltophilia*	4 (4%) [3]
Change in weight z-score, *n* = 28, median (range)	0.39 (−2.00, 1.61) *
FEV1 percent predicted at 7 years, *n* = 23, median (range)	104 (89–138)

*signed rank test *p*-value <0.05

### Growth parameters

Growth parameters improved in this cohort over the 2 year study period. At the time of enrollment, the mean (SE, CI) weight z-score was −0.48 (0.16, −2.29 to 0.91) and increased by a mean (SE) of 0.44 (0.14)). There was no association between the frequency of PEx and BMI (*R* = 0.10), weight (*R* = −0.09) or height (*R* = −0.11) at enrollment. Additionally, there was no association between highest percent predicted FEV_1_ values and height (*R* = 0.05) and therefore FEV_1_ did not appear to be artifactually elevated due to stunting.

### Oropharyngeal culture results

OP swabs were obtained at routine clinic visits and during exacerbation visits. Methicillin sensitive *S. aureus* (MSSA) and *Haemophilus influenzae* were the most common pathogens identified on OP cultures. *Pseudomonas aeruginosa* was identified in four subjects and none were chronically infected. One subject was hospitalized due to a pulmonary exacerbation at the time of the new *Pseudomonas* infection and received 2 weeks of IV antibiotics followed by 1 month of inhaled tobramycin. This subject was hospitalized a total of 3 times during the study. The remaining subjects were treated with 4 weeks of inhaled tobramycin for *Pseudomonas* eradication and one of these three subjects also received 2 weeks of oral ciprofloxacin. None of the subjects treated for *Pseudomonas* on an outpatient basis were hospitalized during the two-year study period. The proportion of positive *H. influenzae* cultures was modestly associated with lower PEx frequency [*R* = −0.31, (−0.60, 0.06)] but not FEV_1_ at age 7 years (*R* = −0.26,). No correlation was seen between the proportion of positive MSSA cultures with exacerbation frequency (*R* = −0.01) or FEV_1_ values (*R* = 0.12).

### Pulmonary function testing

**Fig. 3:**
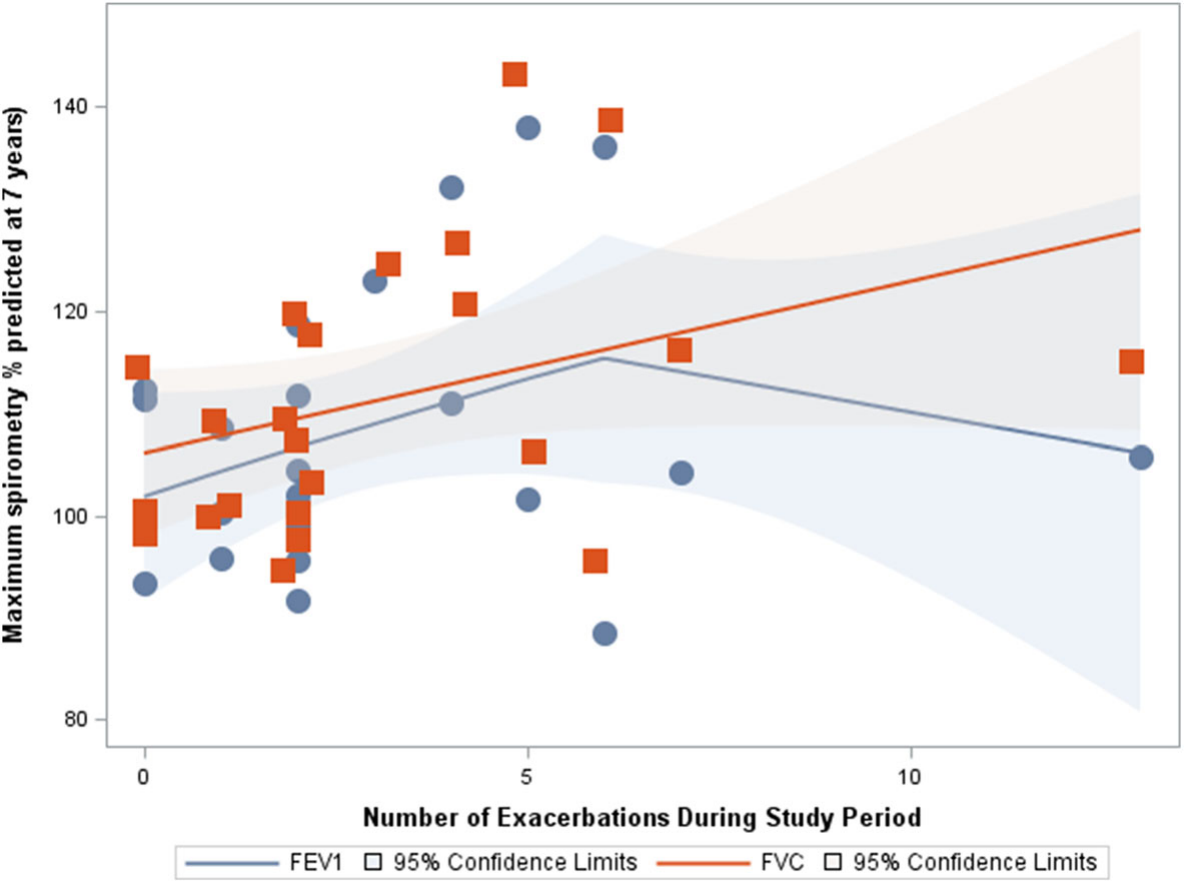
Relationship between lung function at 7 years and number of exacerbations: A loess curve for FEV1 (blue line) and FVC (red line) was used to display the association and is plotted with a 95% confidence interval (shaded bands). Higher FVC was associated with increased exacerbation frequency whereas no association was seen between FEV1 and exacerbation frequency

Pulmonary function testing between 6.5 and 7.5 years was performed in 23 out of 30 patients. Additional details including absolute, z-score and percent predicted values of FEV1, FVC and FEV1/FVC for each individual subject are included in the data supplement (See Additional file 3: Table S2). The remaining subjects have not yet reached 6.5 years of age and 4 subjects withdrew from the study and therefore this data was not assessed. The median FEV_1_ in this group was 104% (z-score 0.65, 95% CI: 0.16–1.14) with a range of 89–138% predicted. Lung function was not associated with gender, history of meconium ileus, weight z-score or the baseline chest radiograph score. Higher FVC values were associated with more frequent exacerbations [*R* = 0.44, (0.03, 0.72)] while FEV_1_ was not associated with exacerbation frequency [*R* = 0.20, (−0.23, 0.57)] (Fig. 3). Higher FEV_1_ was associated with lower Wisconsin cough score at enrollment [*R* = −0.48, (−0.76, −0.04)], (Additional file 4: Figure S2). Pulmonary function testing is variable in this age group and therefore we selected the highest percent predicted FEV1 between 6.5 and 7.5 years as a marker for lung function at 7 years of age. A strong correlation existed in this cohort between the highest FEV_1_ and mean FEV_1_ values (data not shown) and therefore use of the mean FEV_1_ in these calculations would be unlikely to change the results.

The association observed between FEV_1_ and exacerbation frequency was used to estimate a sample size needed to detect this correlation in a future study. A sample size of 193 would be required to detect a correlation of 0.20 between the number of pre-school exacerbations and FEV_1_ at age 7 with 80% power using a two-sided hypothesis test with a 0.05 significance level.

## Discussion

We found in this pilot study that PEx were common in young children with CF, with the vast majority of children experiencing more than one PEx over a 2 year period. Although most exacerbations did not require hospitalization, over a quarter of the children were hospitalized at least once during the study. Importantly, this study also demonstrates that longitudinal studies of exacerbations of children with cystic fibrosis are feasible as 26/30 (87%) completed the study. We found that lower chest radiograph scores, suggesting more structural lung damage, were associated with an increased number of exacerbations in the subsequent 2 years. Contrary to our hypothesis, we did not find an association between exacerbation frequency and lower FEV1 and FVC values.

This study is significant because it demonstrates that chest radiograph scores may be predictive of an individual’s risk for future exacerbations. Other studies have also demonstrated that chest radiographs may have value in monitoring the progression of CF lung disease [18]. Spirometry is a less sensitive marker of structural damage in mild disease [19]; thus, particularly in young children, it may be that abnormalities in spirometry will only become apparent after longer follow up times. This may explain why the number of exacerbations was associated with worse chest x-ray scores but not with spirometry values. A potentially more sensitive marker of lung disease, lung clearance index (LCI) has been compared to spirometry including FEV1 in several studies. Abnormalities in LCI are detected earlier than abnormalities in FEV_1_ [20–23]_._ Worsening LCI measurements over time have been shown in young children with CF compared to healthy controls, whereas, no difference in the rate of decline in FEV_1_ compared to healthy controls was found [24]. These studies suggest that LCI may be a more sensitive marker of early disease progression compared to FEV_1_ in young children with CF.

This study also provides information on the relationship between PEx, antibiotic frequency and later pulmonary function. We speculate that spirometry values were not lower in the setting of an increased number of PEx due to the increased use of antibiotic therapy in these patients. This suggests that frequent use of antibiotics in patients with increased respiratory symptoms may be beneficial. In contrast to our findings, Byrnes and colleagues demonstrated that exacerbations in the first 2 years of life were associated with lower FEV1 values at age 5 [25]*.* Our cohort differs from the Australian cohort where a higher rate of prophylactic antibiotics and *Pseudomonas aeruginosa* infections were noted [25]. The increased use of prophylactic antibiotics in this cohort may have impacted both exacerbation frequency and later lung function contributing to the differences in findings. Ramsey and colleagues noted that patients who received prophylactic antibiotics in the first 2 years of life had higher FEV_0.75_ and FVC compared to those who did not receive them [26].

Regelmann and investigators of the Epidemiologic Study of Cystic Fibrosis (ESCF) assessed the association between clinical findingsand exacerbation frequency and the impact of treatment for an exacerbation on FEV_1_ percent predicted at age 7 years [27]. They concluded that patients with a higher number of clinical characteristics had lower FEV_1_ percent predicted at 7 years and that treatment did not impact later FEV_1_ [27]. There are key differences that exist with our study. Specifically, the percentage of patients with *P. aeruginosa* was markedly higher in the study by Regelmann and coworkers [27]. This study also only captured data from one exacerbation over the study period [27], whereas we evaluated all outpatient and inpatient exacerbations over a 2 year period. Finally, CF care has evolved as our cohort was enrolled later than the ESCF cohort.

The effect of antibiotics on lung function has been evaluated in older children and adults but less is known in younger children. A study performed by Schechter and coworkers through ESCF, evaluated pediatric care centers and compared median FEV_1_ percent predicted and frequency of antibiotic prescriptions for PEx Centers with the highest quartile for FEV_1_ were more likely to prescribe antibiotics for signs of a PEx [28]. Although this may not be the only factor for higher FEV_1_ values in these centers, the authors felt that it was likely a contributor to better lung function [28]. Stanojevic and coworkers performed a retrospective study of 570 adult and pediatric CF patients (median age of 21 years) and assessed the impact of exacerbations treated with oral antibiotics on lung function [29]. They determined that decline in FEV1 was greater in those with more frequent exacerbations [29]. Although their models were adjusted for age and baseline lung function, it is unclear if the results would be the same in a younger population with less severe lung disease and an earlier age of diagnosis.

Limitations of our study included enrolling a relatively small number of subjects. Larger studies are needed to better define the relationship between early exacerbations and later pulmonary function. However, to best capture exacerbations, prospective studies are likely needed as outpatient exacerbations treated with oral antibiotics are not well captured in the registry. Additionally, the number of subjects experiencing more severe exacerbations requiring hospitalization was small and may limit our ability to ascertain an effect on clinical outcomes. The wide age range of patients at the time of enrollment is also a limitation. Although this study was small, the results may inform study size and design for larger clinical trials related to PEx in young children and infants.

The use of clinician defined exacerbations, instead of using a standardized exacerbation definition, could be considered a limitation of this study, although no standardized definition exists for this age range. Based on the retrospective analysis of our patients’ symptoms during an exacerbation, all but one clinically defined exacerbation would also meet criteria for an exacerbation based on a previously used exacerbation definition [12]. However, additional studies are needed to better define PEx in infants and young children with CF.

## Conclusions

In this pilot study of 30 young children with CF we found that exacerbations are common and that worse chest x-ray scores, both at enrollment and study completion, were associated with increased PEx. Spirometry values (FEV_1_ and FVC) were not lower in patients with more frequent exacerbations, contrary to our hypothesis, suggesting treatment with antibiotics in the setting of increased respiratory symptoms is beneficial. However, spirometry in this age range may not be the most sensitive marker for mild lung disease or disease progression. Additional studies are indicated in order to better understand the impact of pulmonary exacerbations, oral antibiotic treatment and the effect on lung function. Furthermore, our study provides data with respect to chest x-ray score, frequency of exacerbations and the relationship between exacerbations, antibiotic use and lung function that could be used in power analyses for larger studies.

## Additional files

Additional file 1: Figure S1. Study Design: 30 subjects with CF were enrolled in the study. Study visits were performed at quarterly CF visits and at the time of an exacerbation over a two-year period. At each study visit, subjects underwent a history, physical, medication history and a culture obtained by oropharyngeal (OP) swab. Chest radiographs were done at study enrollment and at study completion (2 years) during periods of clinical stability and assigned a Brasfield score. (JPEG 27 kb)

Additional file 2: Table S1. Components of Chest X-ray Score for Individual Subjects: Components of the chest x-ray score at study enrollment and study completion are provided for each individual subject. (DOCX 32 kb)

Additional file 3: Table S2. Pulmonary function testing results: Absolute, z-score and percent predicted values of FEV1, FVC and FEV1/FVC are provided for each subject. (DOCX 14 kb)

Additional file 4: Figure S2. Relationship between lung function at 7 years and Wisconsin cough score at enrollment: A loess curve (grey line) was used to display the association and is plotted with a 95% confidence interval (shaded band). Higher FEV1 percent predicted at school age was associated with a lower Wisconsin cough score at enrollment. The corresponding linear association for the rank transformed variables is indicated by the Spearman’s rank-based correlation coefficient in the upper right hand corner. (PDF 83 kb)

## Abbreviations

CF: Cystic fibrosis
CXR: Chest x-ray
ESCF: Epidemiologic Study of Cystic Fibrosis
FEV1: Forced expiratory volume in 1 second
FVC: Forced vital capacity
GLI: Global Lung Function Initiative
IV: Intravenous
LCI: Lung clearance index
MSSA: Methicillin sensitive *Staphylococcus aureus*
OP: Oropharyngeal
PEx: Pulmonary exacerbations
