# Supplementary figures and images for: Pulmonary exacerbations and clinical outcomes in a longitudinal cohort of infants and preschool children with cystic fibrosis
Source: BMC Pulm Med. 2017 Dec 11;17:188. doi: 10.1186/s12890-017-0546-8 (PMC5725640; doi:10.1186/s12890-017-0546-8)

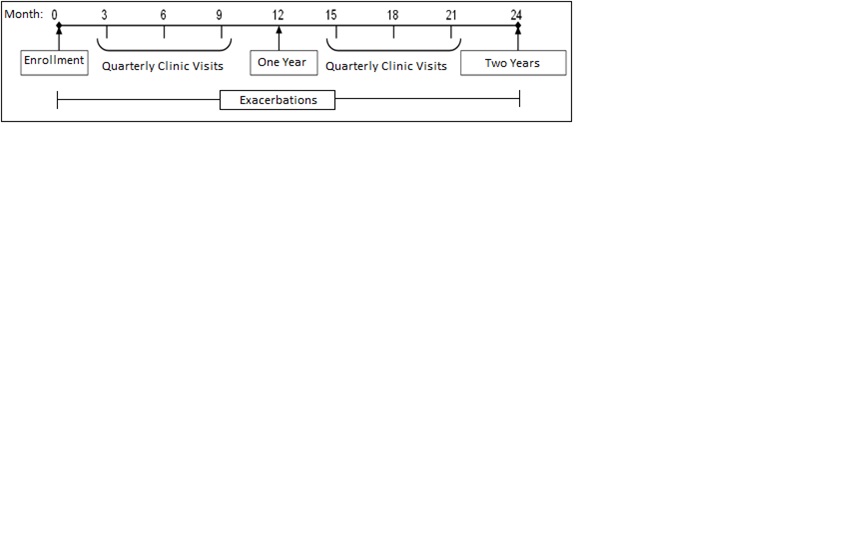

Supplement: Supplementary file 1 — Study Design: 30 subjects with CF were enrolled in the study. Study visits were performed at quarterly CF visits and at the time of an exacerbation over a two-year period. At each study visit, subjects underwent a history, physical, medication history and a culture obtained by oropharyngeal (OP) swab. Chest radiographs were done at study enrollment and at study completion (2 years) during periods of clinical stability and assigned a Brasfield score. (JPEG 27 kb) [file 12890_2017_546_MOESM1_ESM.jpg]

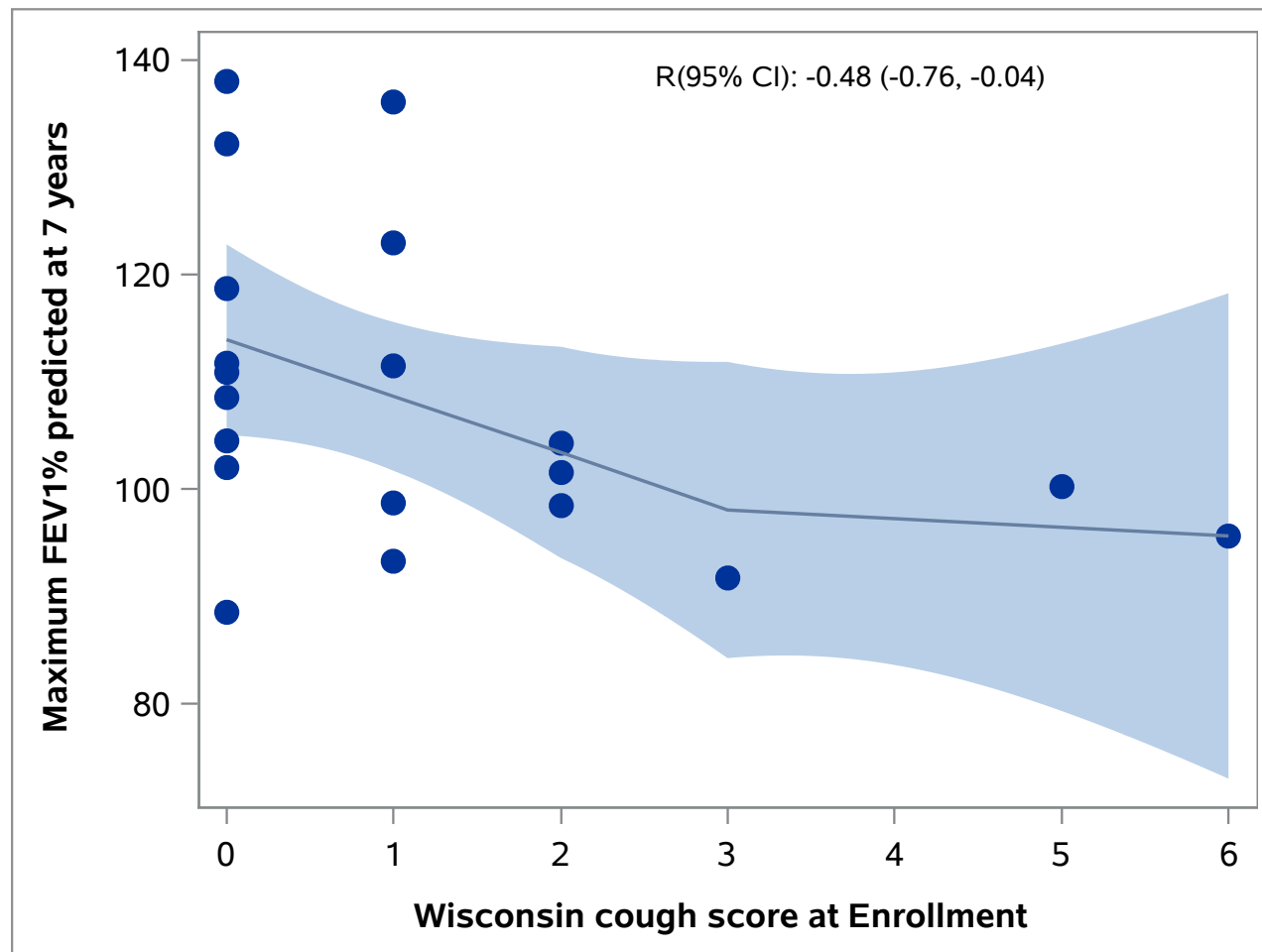

Supplement: Supplementary file 4 — Relationship between lung function at 7 years and Wisconsin cough score at enrollment: A loess curve (grey line) was used to display the association and is plotted with a 95% confidence interval (shaded band). Higher FEV1 percent predicted at school age was associated with a lower Wisconsin cough score at enrollment. The corresponding linear association for the rank transformed variables is indicated by the Spearman’s rank-based correlation coefficient in the upper right hand corner. (PDF 83 kb) [file 12890_2017_546_MOESM4_ESM.pdf]
