# Supplementary material for: Pulmonary exacerbations and clinical outcomes in a longitudinal cohort of infants and preschool children with cystic fibrosis
Source: BMC Pulm Med. 2017 Dec 11;17:188. doi: 10.1186/s12890-017-0546-8 (PMC5725640; doi:10.1186/s12890-017-0546-8)
Supplement: Supplementary file 2 — Components of Chest X-ray Score for Individual Subjects: Components of the chest x-ray score at study enrollment and study completion are provided for each individual subject. (DOCX 32 kb) [file 12890_2017_546_MOESM2_ESM.docx]

Additional file 2: Table S1: Components of Chest X-ray Score for Individual Subjects

| **Subject** | **Reader** | **Time Point** | **Air trapping** | **Linear markings** | **Nodular cystic lesions** | **Large lesions** | **General severity** | **Sum** | **Score** |
| --- | --- | --- | --- | --- | --- | --- | --- | --- | --- |
| 001 | 1 | Initial | 0 | 0 | 0 | 0 | 0 | 0 | 25 |
|  | 2 | Initial | 0 | 1 | 0 | 0 | 1 | 2 | 23 |
|  | 1 | Study Completion | 1 | 2 | 0 | 0 | 1 | 4 | 21 |
|  | 2 | Study Completion | 1 | 2 | 0 | 0 | 1 | 4 | 21 |
| 002 | 1 | Initial | 0 | 1 | 0 | 0 | 1 | 2 | 23 |
|  | 2 | Initial | 1 | 2 | 1 | 0 | 2 | 6 | 19 |
|  | 1 | Study Completion | 0 | 2 | 0 | 0 | 2 | 4 | 21 |
|  | 2 | Study Completion | 1 | 2 | 0 | 0 | 2 | 5 | 20 |
| 003 | 1 | Initial | 0 | 1 | 0 | 0 | 1 | 2 | 23 |
|  | 2 | Initial | 1 | 2 | 1 | 0 | 2 | 6 | 19 |
|  | 1 | Study Completion | 0 | 2 | 1 | 0 | 2 | 5 | 20 |
|  | 2 | Study Completion | 0 | 2 | 0 | 0 | 1 | 3 | 22 |
| 004 | 1 | Initial | 0 | 1 | 0 | 0 | 1 | 2 | 23 |
|  | 2 | Initial | 0 | 2 | 0 | 0 | 1 | 3 | 22 |
|  | 1 | Study Completion | 0 | 2 | 0 | 0 | 2 | 4 | 21 |
|  | 2 | Study Completion | 0 | 2 | 0 | 0 | 2 | 4 | 21 |
| 005 | 1 | Initial | 0 | 2 | 0 | 0 | 1 | 3 | 22 |
|  | 2 | Initial | 0 | 1 | 0 | 0 | 1 | 2 | 23 |
|  | 1 | Study Completion | 0 | 2 | 0 | 0 | 1 | 3 | 22 |
|  | 2 | Study Completion | 0 | 1 | 0 | 0 | 0 | 1 | 24 |
| 006 | 1 | Initial | 0 | 2 | 0 | 0 | 1 | 3 | 22 |
|  | 2 | Initial | 1 | 2 | 0 | 0 | 2 | 5 | 20 |
|  | 1 | Study Completion | 0 | 2 | 0 | 0 | 2 | 4 | 21 |
|  | 2 | Study Completion | 2 | 2 | 1 | 0 | 2 | 7 | 18 |
| 007 | 1 | Initial | 0 | 1 | 0 | 0 | 1 | 2 | 23 |
|  | 2 | Initial | 0 | 2 | 1 | 0 | 1 | 4 | 21 |
|  | 1 | Study Completion | 0 | 2 | 0 | 0 | 1 | 3 | 22 |
|  | 2 | Study Completion | 0 | 2 | 0 | 0 | 2 | 4 | 21 |
| 008 | 1 | Initial | 0 | 1 | 0 | 0 | 1 | 2 | 23 |
|  | 2 | Initial | 0 | 1 | 0 | 0 | 1 | 2 | 23 |
| 009 | 1 | Initial | 1 | 1 | 0 | 0 | 1 | 3 | 22 |
|  | 2 | Initial | 2 | 0 | 0 | 0 | 1 | 3 | 22 |
| 010 | 1 | Initial | 0 | 1 | 0 | 0 | 1 | 2 | 23 |
|  | 2 | Initial | 1 | 2 | 0 | 0 | 2 | 5 | 20 |
|  | 1 | Study Completion | 0 | 2 | 0 | 0 | 1 | 3 | 22 |
|  | 2 | Study Completion | 1 | 2 | 2 | 0 | 2 | 7 | 18 |
| 011 | 1 | Initial | 0 | 2 | 0 | 0 | 1 | 3 | 22 |
|  | 2 | Initial | 0 | 1 | 0 | 0 | 1 | 2 | 23 |
|  | 1 | Study Completion | 0 | 1 | 0 | 0 | 1 | 2 | 23 |
|  | 2 | Study Completion | 2 | 1 | 0 | 0 | 1 | 4 | 21 |
| 012 | 1 | Initial | 0 | 1 | 0 | 0 | 1 | 2 | 23 |
|  | 2 | Initial | 0 | 2 | 0 | 0 | 1 | 3 | 22 |
|  | 1 | Study Completion | 0 | 2 | 2 | 0 | 2 | 6 | 19 |
|  | 2 | Study Completion | 0 | 2 | 0 | 0 | 1 | 3 | 22 |
| 013 | 1 | Initial | 0 | 1 | 0 | 0 | 1 | 2 | 23 |
|  | 2 | Initial | 1 | 0 | 0 | 0 | 1 | 2 | 23 |
|  | 1 | Study Completion | 0 | 2 | 0 | 0 | 1 | 3 | 22 |
|  | 2 | Study Completion | 0 | 1 | 0 | 0 | 1 | 2 | 23 |
| 014 | 1 | Initial | 0 | 1 | 0 | 0 | 1 | 2 | 23 |
|  | 2 | Initial | 1 | 3 | 1 | 0 | 3 | 8 | 17 |
|  | 1 | Study Completion | 0 | 2 | 0 | 0 | 2 | 4 | 21 |
|  | 2 | Study Completion | 1 | 2 | 0 | 0 | 2 | 5 | 20 |
| 015 | 1 | Initial | 1 | 3 | 0 | 0 | 2 | 6 | 19 |
|  | 2 | Initial | 2 | 1 | 0 | 0 | 1 | 4 | 21 |
|  | 1 | Study Completion | 0 | 2 | 1 | 0 | 2 | 5 | 20 |
|  | 2 | Study Completion | 0 | 2 | 0 | 0 | 1 | 3 | 22 |
| 016 | 1 | Initial | 1 | 1 | 1 | 0 | 2 | 5 | 20 |
|  | 2 | Initial | 1 | 1 | 0 | 0 | 0 | 2 | 23 |
| 017 | 1 | Initial | 1 | 1 | 1 | 0 | 2 | 5 | 20 |
|  | 2 | Initial | 1 | 1 | 0 | 0 | 1 | 3 | 22 |
|  | 1 | Study Completion | 2 | 1 | 1 | 0 | 1 | 5 | 20 |
|  | 2 | Study Completion | 2 | 2 | 0 | 0 | 2 | 6 | 19 |
| 018 | 1 | Initial | 0 | 1 | 0 | 0 | 1 | 2 | 23 |
|  | 2 | Initial | 1 | 1 | 1 | 0 | 2 | 5 | 20 |
|  | 1 | Study Completion | 0 | 2 | 1 | 0 | 2 | 5 | 20 |
|  | 2 | Study Completion | 1 | 2 | 1 | 0 | 2 | 6 | 19 |
| 019 | 1 | Initial | 0 | 1 | 0 | 0 | 1 | 2 | 23 |
|  | 2 | Initial | 0 | 0 | 0 | 0 | 0 | 0 | 25 |
|  | 1 | Study Completion | 0 | 1 | 0 | 0 | 1 | 2 | 23 |
|  | 2 | Study Completion | 0 | 1 | 0 | 0 | 1 | 2 | 23 |
| 020 | 1 | Initial | 0 | 2 | 0 | 0 | 2 | 4 | 21 |
|  | 2 | Initial | 1 | 2 | 0 | 0 | 2 | 5 | 20 |
|  | 1 | Study Completion | 1 | 1 | 1 | 0 | 2 | 5 | 20 |
|  | 2 | Study Completion | 1 | 2 | 0 | 0 | 1 | 4 | 21 |
| 021 | 1 | Initial | 0 | 2 | 0 | 0 | 1 | 3 | 22 |
|  | 2 | Initial | 1 | 2 | 1 | 0 | 2 | 6 | 19 |
|  | 1 | Study Completion | 0 | 2 | 0 | 0 | 2 | 4 | 21 |
|  | 2 | Study Completion | 0 | 2 | 0 | 0 | 1 | 3 | 22 |
| 022 | 1 | Initial | 0 | 2 | 0 | 0 | 2 | 4 | 21 |
|  | 2 | Initial | 1 | 1 | 2 | 0 | 2 | 6 | 19 |
|  | 1 | Study Completion | 1 | 2 | 2 | 0 | 3 | 8 | 17 |
|  | 2 | Study Completion | 1 | 2 | 2 | 0 | 3 | 8 | 17 |
| 023 | 1 | Initial | 1 | 1 | 0 | 0 | 1 | 3 | 22 |
|  | 2 | Initial | 1 | 1 | 0 | 0 | 1 | 3 | 22 |
|  | 1 | Study Completion | 0 | 2 | 0 | 0 | 1 | 3 | 22 |
|  | 2 | Study Completion | 1 | 1 | 0 | 0 | 1 | 3 | 22 |
| 024 | 1 | Initial | 1 | 2 | 2 | 0 | 3 | 8 | 17 |
|  | 2 | Initial |  | 3 | 0 | 0 | 2 | 5 | 20 |
|  | 1 | Study Completion | 0 | 2 | 0 | 0 | 1 | 3 | 22 |
|  | 2 | Study Completion | 0 | 2 | 0 | 0 | 1 | 3 | 22 |
| 025 | 1 | Initial | 0 | 0 | 0 | 0 | 0 | 0 | 25 |
|  | 2 | Initial | 0 | 0 | 0 | 0 | 0 | 0 | 25 |
|  | 1 | Study Completion | 0 | 1 | 0 | 0 | 1 | 2 | 23 |
|  | 2 | Study Completion | 1 | 1 | 1 | 0 | 1 | 4 | 21 |
| 026 | 1 | Initial | 0 | 0 | 0 | 0 | 0 | 0 | 25 |
|  | 2 | Initial | 0 | 1 | 0 | 0 | 1 | 2 | 23 |
|  | 1 | Study Completion | 0 | 0 | 0 | 0 | 0 | 0 | 25 |
|  | 2 | Study Completion | 0 | 1 | 0 | 0 | 1 | 2 | 23 |
| 028 | 1 | Initial | 0 | 1 | 0 | 0 | 1 | 2 | 23 |
|  | 2 | Initial | 1 | 1 | 0 | 0 | 2 | 4 | 21 |
| 029 | 1 | Initial | 0 | 1 | 1 | 0 | 2 | 4 | 21 |
|  | 2 | Initial | 0 | 1 | 0 | 0 | 1 | 2 | 23 |
|  | 1 | Study Completion | 0 | 3 | 0 | 0 | 2 | 5 | 20 |
|  | 2 | Study Completion | 1 | 3 | 1 | 0 | 2 | 7 | 18 |
| 030 | 1 | Initial | 0 | 2 | 0 | 0 | 1 | 3 | 22 |
|  | 2 | Initial | 0 | 2 | 1 | 0 | 2 | 5 | 20 |
|  | 1 | Study Completion | 0 | 2 | 0 | 0 | 1 | 3 | 22 |
|  | 2 | Study Completion | 1 | 2 | 2 | 0 | 3 | 8 | 17 |
