# Supplementary material for: Pulmonary exacerbations and clinical outcomes in a longitudinal cohort of infants and preschool children with cystic fibrosis
Source: BMC Pulm Med. 2017 Dec 11;17:188. doi: 10.1186/s12890-017-0546-8 (PMC5725640; doi:10.1186/s12890-017-0546-8)
Supplement: Supplementary file 3 — Pulmonary function testing results: Absolute, z-score and percent predicted values of FEV1, FVC and FEV1/FVC are provided for each subject. (DOCX 14 kb) [file 12890_2017_546_MOESM3_ESM.docx]

Additional file 3: Table S2: Pulmonary function testing results

| **SID** | **Age** | **FEV1** | | | **FVC** | | | **FEV1/FVC** | | | **# of PEx** |
| --- | --- | --- | --- | --- | --- | --- | --- | --- | --- | --- | --- |
|  |  | **FEV1** | **z-score** | **Percent**  **predicted** | **FVC** | **z-score** | **Percent**  **predicted** | **FEV1/FVC** | **z-score** | **Percent**  **predicted** |  |
| 001 | 6.8 | 1.67 | 0.68 | 108.5 | 1.91 | 0.73 | 109.2 | 0.87 | -0.22 | 98.50 | 1 |
| 002 | 7.4 | 1.36 | 0.02 | 100.2 | 1.54 | 0.08 | 101.0 | 0.88 | -0.19 | 98.72 | 1 |
| 003 | 7.0 | 1.53 | 0.35 | 104.2 | 1.90 | 1.27 | 116.1 | 0.81 | -1.53 | 89.11 | 7 |
| 004 | 7.4 | 1.75 | 2.61 | 132.2 | 1.87 | 2.26 | 126.7 | 0.94 | 0.65 | 103.9 | 4 |
| 005 | 7.2 | 1.48 | 1.84 | 123.0 | 1.66 | 2.08 | 124.7 | 0.89 | -0.28 | 98.24 | 3 |
| 006 | 6.8 | 1.08 | -0.91 | 88.55 | 1.30 | -0.34 | 95.71 | 0.83 | -1.07 | 92.05 | 6 |
| 007 | 7.4 | 1.53 | 0.36 | 104.5 | 1.78 | 0.60 | 107.4 | 0.86 | -0.48 | 96.62 | 2 |
| 008 | 7.0 | 1.32 | -0.35 | 95.70 | 1.53 | -0.01 | 99.92 | 0.86 | -0.76 | 95.18 | 1 |
| 009 | 7.0 | 1.34 | 0.88 | 112.3 | 1.34 | 0.04 | 100.6 | 1.00 | 1.97 | 111.1 | 0 |
| 010 | 7.0 | 1.53 | 0.91 | 110.9 | 1.85 | 1.63 | 120.8 | 0.83 | -1.28 | 91.24 | 4 |
| 011 | 6.8 | 1.49 | -0.34 | 95.69 | 1.73 | -0.18 | 97.68 | 0.86 | -0.42 | 97.10 | 2 |
| 012 | 7.0 | 1.37 | 0.17 | 102.0 | 1.63 | 0.75 | 109.5 | 0.84 | -1.11 | 92.59 | 2 |
| 013 | 7.1 | 1.46 | -0.54 | 93.30 | 1.75 | -0.14 | 98.25 | 0.83 | -0.80 | 94.18 | 0 |
| 014 | 6.6 | 1.38 | 0.96 | 111.7 | 1.63 | 1.53 | 119.8 | 0.85 | -1.10 | 92.73 | 2 |
| 015 | 6.6 | 1.22 | -0.67 | 91.72 | 1.52 | 0.25 | 103.2 | 0.80 | -1.62 | 88.26 | 2 |
| 017 | 7.1 | 1.77 | 3.22 | 138.0 | 2.03 | 3.35 | 143.3 | 0.87 | -0.66 | 95.85 | 5 |
| 018 | 6.6 | 1.20 | -0.10 | 98.75 | 1.28 | -0.42 | 94.67 | 0.94 | 0.59 | 103.7 | 2 |
| 019 | 7.0 | 1.45 | -0.12 | 98.50 | 1.67 | 0.02 | 100.2 | 0.87 | -0.35 | 97.57 | 2 |
| 020 | 7.0 | 2.08 | 3.05 | 136.1 | 2.37 | 2.99 | 138.8 | 0.88 | -0.44 | 97.32 | 6 |
| 022 | 7.2 | 1.45 | 0.13 | 101.5 | 1.69 | 0.51 | 106.4 | 0.86 | -0.80 | 94.85 | 5 |
| 023 | 6.6 | 1.65 | 1.49 | 118.7 | 1.84 | 1.39 | 117.6 | 0.90 | 0.03 | 100.2 | 2 |
| 025 | 7.3 | 1.57 | 0.93 | 111.4 | 1.82 | 1.17 | 114.5 | 0.86 | -0.47 | 96.69 | 0 |
| 029 | 6.8 | 1.64 | 0.45 | 105.7 | 2.03 | 1.19 | 115.0 | 0.81 | -1.19 | 91.06 | 13 |
